# Acetato­(*N*-[(*E*)-1-(6-methyl-2-pyrid­yl)methyl­idene]-2-{2-[(*E*)-1-(6-methyl-2-pyrid­yl)methyl­idene­amino]­pheneth­yl}aniline)nickel(II) perchlorate

**DOI:** 10.1107/S1600536810034446

**Published:** 2010-09-04

**Authors:** Shuranjan Sarkar, Hajin Lee, Hong-In Lee

**Affiliations:** a Department of Chemistry, Kyungpook National University, Daegu, 702-701, Republic of Korea; b Korea Basic Science Institute, 664-14 Dukjin dong 1-ga, Dukjin-gu, Jeonju, 561-756, Republic of Korea

## Abstract

In the title complex, [Ni(CH_3_COO)(C_28_H_26_N_4_)]ClO_4_, the Ni^II^ atom is coordinated by two imine N atoms and two pyridine N atoms of the *N*-[(*E*)-1-(6-methyl-2-pyrid­yl)methyl­idene]-2-(2-[(*E*)-1-(6-methyl-2-pyrid­yl)methyl­idene­amino]­pheneth­yl)aniline donor ligand and two O atoms of the acetate ion in a distorted octa­hedral coordination. The average Ni—N and Ni—O bond lengths are 2.131 (13) and 2.098 (11) Å, respectively. An intramolecular N—H⋯O inter­action occurs. Relatively weak inter­molecular C—H⋯O inter­actions between the ligands and the ClO_4_^−^ ions result in a chain extending along the *b* axis.

## Related literature

For structures of Ni complexes with ligands formed by the condensation of 2-pyridyl aldehydes and a variety of diamines, see: Banerjee *et al.* (2004 ▶). For comparison Ni—N bond distances, see: Martin *et al.* (1977 ▶).
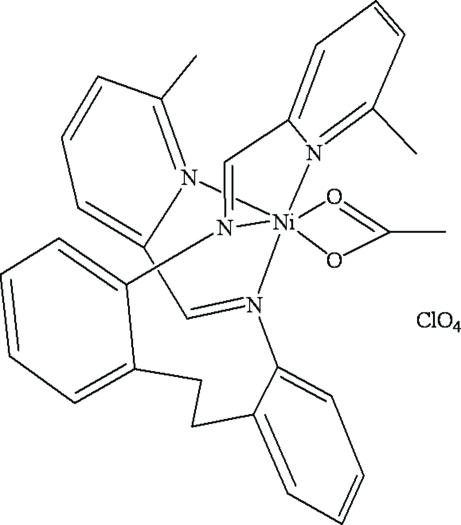


## Experimental

### 

#### Crystal data

[Ni(C_2_H_3_O_2_)(C_28_H_26_N_4_)]ClO_4_
*M* *_r_* = 635.73
Triclinic, 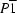

*a* = 8.5759 (8) Å
*b* = 11.4975 (10) Å
*c* = 14.8322 (13) Å
α = 79.392 (2)°
β = 78.102 (2)°
γ = 81.327 (2)°
*V* = 1396.9 (2) Å^3^
*Z* = 2
Mo *K*α radiation
μ = 0.84 mm^−1^
*T* = 200 K
0.23 × 0.11 × 0.10 mm

#### Data collection

Bruker APEX CCD area-detector diffractometer
10545 measured reflections
6856 independent reflections
3311 reflections with *I* > 2σ(*I*)
*R*_int_ = 0.049

#### Refinement

*R*[*F* ^2^ > 2σ(*F* ^2^)] = 0.068
*wR*(*F* ^2^) = 0.149
*S* = 1.05
6856 reflections
382 parameters
H-atom parameters constrained
Δρ_max_ = 1.04 e Å^−3^
Δρ_min_ = −1.84 e Å^−3^

### 

Data collection: *SMART* (Bruker, 2000 ▶); cell refinement: *SAINT* (Bruker, 2000 ▶); data reduction: *SAINT*; program(s) used to solve structure: *SHELXS97* (Sheldrick, 2008 ▶); program(s) used to refine structure: *SHELXL97* (Sheldrick, 2008 ▶); molecular graphics: *SHELXTL* (Sheldrick, 2008 ▶); software used to prepare material for publication: *SHELXTL*.

## Supplementary Material

Crystal structure: contains datablocks I, global. DOI: 10.1107/S1600536810034446/pv2321sup1.cif

Structure factors: contains datablocks I. DOI: 10.1107/S1600536810034446/pv2321Isup2.hkl

Additional supplementary materials:  crystallographic information; 3D view; checkCIF report

## Figures and Tables

**Table 1:** Hydrogen-bond geometry (Å, °)

*D*—H⋯*A*	*D*—H	H⋯*A*	*D*⋯*A*	*D*—H⋯*A*
C4—H4⋯O1^i^	0.95	2.45	3.274 (6)	144
C7—H7⋯O4^ii^	0.95	2.41	3.290 (7)	155
C20—H20⋯O3^iii^	0.95	2.43	3.366 (8)	168
C15—H15*B*⋯O2	0.99	2.54	3.523 (7)	174
C15—H15*B*⋯N3	0.99	2.47	2.935 (7)	108

Symmetry codes: (i) 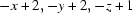; (ii) 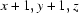; (iii) 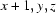.

**Fig. 1.**
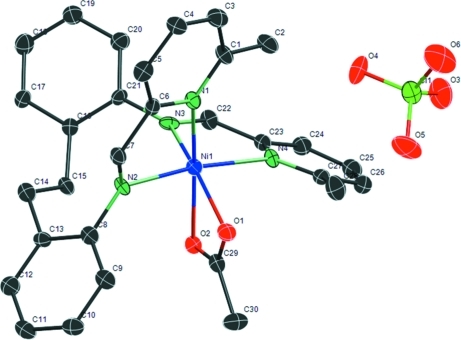
Structure of the title compound showing 33% probability displacement ellipsoids and the atom-numbering scheme; H atoms have been omitted for clarity.

**Fig. 2.**
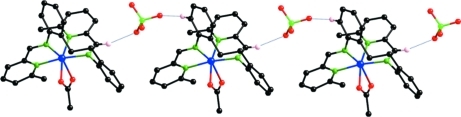
One-dimensional chain of the title complex formed by intermolecular interactions (dotted lines); H atoms have been omitted except for those involved in the C—H···O interactions.

## supplementary crystallographic information

### Comment

The coordination chemistry of Schiff-base ligands formed by the condensation of 2-pyridyl aldehydes with a variety of diamines has been reported recently. These ligands bind tetradentately to metal ions to form a planar arrangement around the metals (Banerjee *et al.*, 2004). We are quite interested in the synthesis of this type of Schiff-base tetraamine nickel(II) complexes and have obtained a novel nickel(II) compound with an acetate group. In this paper, we report the synthesis and crystal structure of the title complex.

The title complex consists of [Ni(mpma)(*η*^2^-CH_3_CO_2_)]^+^ (mpma = N1-[(*E*)-1-(6-methyl-2-pyridyl)-methylidene]-2-(2-[(*E*)-1-(6- methyl-2-pyridyl)methylidene]aminophene-thyl)aniline) cation and ClO^-^_4_ anion (Fig. 1) wherein the Nickel(II) ion is six-coordinated with four N atoms of mpma and two O atoms of the acetate group, giving a distorted octahedral geometry. The average Ni—N and Ni—O bond lengths are 2.131 (13) and 2.098 (11) Å, respectively. It is in good agreement with the general trend that the nickel(II)-nitrogen bonds are longer (or weaker) in the octahedral species than in square planar species (Ni—N = 1.88–1.91 Å) (Martin *et al.*, 1977). The N1—Ni1—N2, N3—Ni1—N4, and O1—Ni1—O2 bond angles are 79.57 (16), 79.87 (18), and 63.16 (14)°, respectively. The deviation of these angles from the ideal octahedral geometry is due to the constraints of the five membered chelate rings (N1—Ni1—N2—C7—C6 and N3—Ni1—N4—N23—N22) and four membered chelate ring (O1—Ni1—O2—C29). The distortion is also reflected in the bond angles N2—Ni1—N4, O2—Ni1—N1, and O1—Ni1—N3, which are 171.81 (19), 164.65 (15), and 158.19 (15)°, respectively. Relatively weak, intermolecular, C—H···O distances are found between the ligand and ClO_4_^-^ ion, forming an one-dimensional chain extended along the *b*-axis (Fig. 2 and Tab. 1).

### Experimental

Nickel(II)acetate tetrahydrate (0.5 g, 2.0 mmol) dissolved in dry methanol (25 ml) was added dropwise to a methanol solution (10 ml) of mpma (0.84 g, 2.0 mmol) and stirred. A green color solution appeared. Then methanol solution (5 ml) of sodium perchlorate (0.25 g, 2.0 mmol) was added. After 1 h, a crystalline powder (1.15 g) was collected by filtration and dried in vacuum. The powder (*ca*. 0.7 g) was dissolved in dry methanol (5 ml) and then diethyl ether (5 ml) was added slowly into the methanol solution. Suitable crystals for X-ray analysis were obtained from the solution after one day.

### Refinement

All H atoms were placed in geometrically idealized positions and constrained to ride on their parent atoms, with distances C—H = 0.95, 0.98 and 0.99 Å for aryl, methyl and methylene H-atoms and *U*_iso_(H) = 1.5 (methyl) and 1.2 (the rest) × the *U*_eq_ of the parent C-atoms. A relatively large residual density on Ni ion is a ghost peak residing less than 1 Å from the ion. An absorption correction did not improve the refinement.

### Crystal data

**Table:**

[Ni(C_2_H_3_O_2_)(C_28_H_26_N_4_)]ClO_4_	*Z* = 2
*M**_r_* = 635.73	*F*(000) = 660
Triclinic, *P*1	*D*_x_ = 1.511 Mg m^−^^3^
Hall symbol: -P 1	Mo *K*α radiation, λ = 0.71073 Å
*a* = 8.5759 (8) Å	Cell parameters from 2599 reflections
*b* = 11.4975 (10) Å	θ = 2.4–26.0°
*c* = 14.8322 (13) Å	µ = 0.84 mm^−^^1^
α = 79.392 (2)°	*T* = 200 K
β = 78.102 (2)°	Block, yellow
γ = 81.327 (2)°	0.23 × 0.11 × 0.10 mm
*V* = 1396.9 (2) Å^3^	

### Data collection

**Table:**

Bruker APEX CCD area-detector diffractometer	3311 reflections with *I* > 2σ(*I*)
Radiation source: fine-focus sealed tube	*R*_int_ = 0.049
graphite	θ_max_ = 28.3°, θ_min_ = 1.4°
φ & ω scans	*h* = −9→11
10545 measured reflections	*k* = −15→15
6856 independent reflections	*l* = −18→19

### Refinement

**Table:**

Refinement on *F*^2^	Primary atom site location: structure-invariant direct methods
Least-squares matrix: full	Secondary atom site location: difference Fourier map
*R*[*F*^2^ > 2σ(*F*^2^)] = 0.068	Hydrogen site location: inferred from neighbouring sites
*wR*(*F*^2^) = 0.149	H-atom parameters constrained
*S* = 1.05	*w* = 1/[σ^2^(*F*_o_^2^) + 2.2621*P*] where *P* = (*F*_o_^2^ + 2*F*_c_^2^)/3
6856 reflections	(Δ/σ)_max_ < 0.001
382 parameters	Δρ_max_ = 1.04 e Å^−^^3^
0 restraints	Δρ_min_ = −1.84 e Å^−^^3^

### Special details

**Table:**

Geometry. All e.s.d.'s (except the e.s.d. in the dihedral angle between two l.s. planes) are estimated using the full covariance matrix. The cell e.s.d.'s are taken into account individually in the estimation of e.s.d.'s in distances, angles and torsion angles; correlations between e.s.d.'s in cell parameters are only used when they are defined by crystal symmetry. An approximate (isotropic) treatment of cell e.s.d.'s is used for estimating e.s.d.'s involving l.s. planes.
Refinement. Refinement of *F*^2^ against ALL reflections. The weighted *R*-factor *wR* and goodness of fit *S* are based on *F*^2^, conventional *R*-factors *R* are based on *F*, with *F* set to zero for negative *F*^2^. The threshold expression of *F*^2^ > σ(*F*^2^) is used only for calculating *R*-factors(gt) *etc*. and is not relevant to the choice of reflections for refinement. *R*-factors based on *F*^2^ are statistically about twice as large as those based on *F*, and *R*- factors based on ALL data will be even larger.

### Fractional atomic coordinates and isotropic or equivalent isotropic displacement parameters (Å^2^)

**Table:**

	*x*	*y*	*z*	*U*_iso_*/*U*_eq_	
Ni1	0.81516 (9)	0.76280 (6)	0.72685 (5)	0.02685 (19)	
N1	1.0115 (5)	0.8119 (4)	0.6208 (3)	0.0245 (10)	
C1	1.1033 (7)	0.7543 (5)	0.5523 (4)	0.0314 (14)	
C2	1.0751 (7)	0.6312 (5)	0.5466 (4)	0.0404 (16)	
H2A	1.0736	0.5815	0.6080	0.061*	
H2B	1.1614	0.5975	0.5010	0.061*	
H2C	0.9719	0.6338	0.5273	0.061*	
C3	1.2207 (7)	0.8104 (5)	0.4861 (4)	0.0382 (16)	
H3	1.2797	0.7698	0.4372	0.046*	
C4	1.2535 (7)	0.9230 (5)	0.4898 (4)	0.0342 (15)	
H4	1.3343	0.9602	0.4447	0.041*	
C5	1.1637 (7)	0.9801 (5)	0.5622 (4)	0.0351 (15)	
H5	1.1824	1.0574	0.5681	0.042*	
C6	1.0472 (7)	0.9219 (5)	0.6249 (4)	0.0283 (13)	
C7	0.9486 (7)	0.9817 (5)	0.7008 (4)	0.0289 (13)	
H7	0.9575	1.0627	0.7020	0.035*	
N2	0.8519 (5)	0.9256 (4)	0.7645 (3)	0.0264 (11)	
C8	0.7531 (7)	0.9948 (5)	0.8311 (4)	0.0294 (14)	
C9	0.6261 (7)	1.0765 (4)	0.8044 (4)	0.0329 (15)	
H9	0.6029	1.0833	0.7436	0.039*	
C10	0.5334 (7)	1.1478 (5)	0.8670 (4)	0.0353 (15)	
H10	0.4468	1.2029	0.8490	0.042*	
C11	0.5679 (7)	1.1381 (5)	0.9551 (4)	0.0373 (16)	
H11	0.5057	1.1872	0.9976	0.045*	
C12	0.6942 (7)	1.0560 (5)	0.9817 (4)	0.0364 (15)	
H12	0.7180	1.0502	1.0423	0.044*	
C13	0.7850 (7)	0.9829 (4)	0.9209 (4)	0.0275 (13)	
C14	0.9193 (7)	0.8913 (5)	0.9504 (4)	0.0347 (15)	
H14A	0.9391	0.9050	1.0109	0.042*	
H14B	1.0188	0.9024	0.9037	0.042*	
C15	0.8821 (7)	0.7607 (4)	0.9605 (4)	0.0290 (13)	
H15A	0.8297	0.7337	1.0254	0.035*	
H15B	0.8075	0.7567	0.9186	0.035*	
C16	1.0336 (7)	0.6814 (4)	0.9361 (4)	0.0252 (12)	
C17	1.1568 (7)	0.6698 (5)	0.9882 (4)	0.0308 (14)	
H17	1.1402	0.7108	1.0401	0.037*	
C18	1.2997 (7)	0.6012 (5)	0.9660 (4)	0.0393 (15)	
H18	1.3804	0.5954	1.0024	0.047*	
C19	1.3277 (8)	0.5402 (5)	0.8913 (4)	0.0418 (16)	
H19	1.4274	0.4930	0.8759	0.050*	
C20	1.2097 (7)	0.5483 (5)	0.8389 (4)	0.0360 (15)	
H20	1.2283	0.5062	0.7876	0.043*	
C21	1.0633 (7)	0.6177 (5)	0.8609 (4)	0.0272 (13)	
N3	0.9426 (5)	0.6226 (4)	0.8063 (3)	0.0253 (11)	
C22	0.9007 (7)	0.5194 (5)	0.8015 (4)	0.0308 (14)	
H22	0.9467	0.4488	0.8354	0.037*	
C23	0.7849 (7)	0.5120 (4)	0.7447 (4)	0.0264 (13)	
C24	0.7234 (7)	0.4057 (5)	0.7518 (4)	0.0359 (15)	
H24	0.7571	0.3367	0.7924	0.043*	
C25	0.6106 (7)	0.4033 (5)	0.6974 (4)	0.0387 (16)	
H25	0.5607	0.3333	0.7032	0.046*	
C26	0.5713 (7)	0.5021 (5)	0.6354 (4)	0.0391 (16)	
H26	0.4949	0.5008	0.5975	0.047*	
C27	0.6460 (7)	0.6062 (5)	0.6284 (4)	0.0296 (13)	
C28	0.6155 (8)	0.7094 (5)	0.5526 (4)	0.0491 (18)	
H28A	0.5150	0.7579	0.5738	0.074*	
H28B	0.6074	0.6790	0.4964	0.074*	
H28C	0.7043	0.7584	0.5384	0.074*	
N4	0.7454 (5)	0.6115 (4)	0.6852 (3)	0.0267 (11)	
O1	0.6159 (5)	0.8708 (3)	0.6811 (3)	0.0337 (10)	
O2	0.5968 (4)	0.7613 (3)	0.8201 (2)	0.0291 (9)	
C29	0.5314 (7)	0.8311 (5)	0.7580 (4)	0.0317 (14)	
C30	0.3545 (7)	0.8655 (5)	0.7740 (4)	0.0455 (17)	
H30A	0.3315	0.9522	0.7700	0.068*	
H30B	0.3063	0.8271	0.8361	0.068*	
H30C	0.3092	0.8397	0.7265	0.068*	
Cl1	0.1830 (2)	0.26066 (14)	0.70157 (11)	0.0417 (4)	
O3	0.2297 (6)	0.3780 (4)	0.6741 (3)	0.0703 (16)	
O4	0.0681 (6)	0.2487 (4)	0.6474 (3)	0.0619 (14)	
O5	0.3214 (6)	0.1768 (4)	0.6830 (3)	0.0754 (17)	
O6	0.1128 (7)	0.2433 (5)	0.7980 (3)	0.0837 (19)	

### Atomic displacement parameters (Å^2^)

**Table:**

	*U*^11^	*U*^22^	*U*^33^	*U*^12^	*U*^13^	*U*^23^
Ni1	0.0292 (4)	0.0244 (4)	0.0265 (4)	−0.0057 (3)	−0.0023 (3)	−0.0036 (3)
N1	0.030 (3)	0.019 (2)	0.024 (2)	−0.006 (2)	−0.003 (2)	−0.0036 (19)
C1	0.044 (4)	0.028 (3)	0.020 (3)	−0.005 (3)	−0.001 (3)	−0.004 (3)
C2	0.045 (4)	0.041 (4)	0.033 (4)	−0.006 (3)	0.005 (3)	−0.011 (3)
C3	0.042 (4)	0.043 (4)	0.026 (3)	−0.002 (3)	0.000 (3)	−0.003 (3)
C4	0.036 (4)	0.029 (3)	0.033 (3)	−0.006 (3)	0.002 (3)	−0.002 (3)
C5	0.035 (4)	0.035 (3)	0.032 (3)	−0.009 (3)	−0.007 (3)	0.006 (3)
C6	0.028 (3)	0.032 (3)	0.027 (3)	−0.008 (3)	−0.005 (3)	−0.008 (3)
C7	0.031 (3)	0.026 (3)	0.032 (3)	−0.008 (3)	−0.004 (3)	−0.009 (3)
N2	0.034 (3)	0.026 (2)	0.020 (2)	−0.005 (2)	0.000 (2)	−0.010 (2)
C8	0.036 (4)	0.020 (3)	0.030 (3)	−0.009 (3)	0.000 (3)	−0.001 (3)
C9	0.042 (4)	0.019 (3)	0.035 (3)	−0.007 (3)	0.001 (3)	−0.001 (3)
C10	0.033 (4)	0.024 (3)	0.044 (4)	−0.002 (3)	0.000 (3)	−0.003 (3)
C11	0.039 (4)	0.031 (3)	0.038 (4)	−0.012 (3)	0.011 (3)	−0.009 (3)
C12	0.036 (4)	0.035 (3)	0.038 (4)	−0.008 (3)	−0.004 (3)	−0.006 (3)
C13	0.025 (3)	0.019 (3)	0.036 (3)	−0.001 (2)	−0.001 (3)	−0.006 (3)
C14	0.039 (4)	0.034 (3)	0.031 (3)	−0.007 (3)	−0.004 (3)	−0.007 (3)
C15	0.034 (3)	0.029 (3)	0.025 (3)	−0.009 (3)	0.001 (3)	−0.009 (3)
C16	0.029 (3)	0.024 (3)	0.023 (3)	−0.008 (2)	−0.004 (2)	0.001 (2)
C17	0.029 (3)	0.032 (3)	0.032 (3)	−0.004 (3)	−0.010 (3)	−0.005 (3)
C18	0.039 (4)	0.040 (4)	0.042 (4)	0.000 (3)	−0.019 (3)	−0.005 (3)
C19	0.036 (4)	0.045 (4)	0.046 (4)	0.007 (3)	−0.014 (3)	−0.013 (3)
C20	0.033 (4)	0.039 (4)	0.036 (4)	−0.002 (3)	−0.008 (3)	−0.006 (3)
C21	0.024 (3)	0.035 (3)	0.023 (3)	−0.004 (3)	−0.009 (2)	0.000 (3)
N3	0.024 (3)	0.026 (2)	0.019 (2)	0.008 (2)	−0.001 (2)	0.002 (2)
C22	0.041 (4)	0.018 (3)	0.033 (3)	−0.008 (3)	−0.006 (3)	−0.001 (2)
C23	0.029 (3)	0.019 (3)	0.029 (3)	−0.007 (2)	0.000 (3)	−0.003 (2)
C24	0.033 (4)	0.039 (4)	0.035 (4)	−0.007 (3)	−0.001 (3)	−0.007 (3)
C25	0.041 (4)	0.031 (3)	0.044 (4)	−0.013 (3)	0.004 (3)	−0.013 (3)
C26	0.039 (4)	0.041 (4)	0.040 (4)	−0.009 (3)	−0.003 (3)	−0.015 (3)
C27	0.026 (3)	0.036 (3)	0.026 (3)	−0.003 (3)	−0.003 (3)	−0.008 (3)
C28	0.065 (5)	0.049 (4)	0.036 (4)	−0.006 (4)	−0.014 (3)	−0.008 (3)
N4	0.033 (3)	0.025 (2)	0.025 (3)	−0.006 (2)	−0.005 (2)	−0.009 (2)
O1	0.036 (3)	0.034 (2)	0.028 (2)	−0.0085 (19)	0.0022 (19)	−0.0041 (19)
O2	0.034 (2)	0.028 (2)	0.024 (2)	−0.0031 (18)	−0.0057 (18)	−0.0012 (17)
C29	0.037 (4)	0.032 (3)	0.028 (3)	−0.006 (3)	−0.005 (3)	−0.009 (3)
C30	0.032 (4)	0.053 (4)	0.045 (4)	0.003 (3)	−0.002 (3)	−0.002 (3)
Cl1	0.0513 (11)	0.0365 (9)	0.0393 (9)	−0.0018 (8)	−0.0136 (8)	−0.0081 (7)
O3	0.092 (4)	0.046 (3)	0.081 (4)	−0.033 (3)	−0.013 (3)	−0.015 (3)
O4	0.067 (4)	0.065 (3)	0.068 (3)	−0.028 (3)	−0.032 (3)	−0.008 (3)
O5	0.083 (4)	0.068 (3)	0.065 (4)	0.045 (3)	−0.022 (3)	−0.020 (3)
O6	0.105 (5)	0.099 (4)	0.032 (3)	0.003 (4)	0.001 (3)	0.003 (3)

### Geometric parameters (Å, °)

**Table:**

Ni1—O2	2.087 (4)	C15—H15A	0.9900
Ni1—O1	2.109 (4)	C15—H15B	0.9900
Ni1—N3	2.117 (4)	C16—C21	1.405 (7)
Ni1—N1	2.121 (4)	C16—C17	1.410 (7)
Ni1—N2	2.133 (4)	C17—C18	1.368 (8)
Ni1—N4	2.151 (4)	C17—H17	0.9500
Ni1—C29	2.417 (6)	C18—C19	1.380 (8)
N1—C6	1.360 (6)	C18—H18	0.9500
N1—C1	1.361 (6)	C19—C20	1.380 (8)
C1—C3	1.396 (7)	C19—H19	0.9500
C1—C2	1.491 (7)	C20—C21	1.394 (7)
C2—H2A	0.9800	C20—H20	0.9500
C2—H2B	0.9800	C21—N3	1.428 (7)
C2—H2C	0.9800	N3—C22	1.309 (6)
C3—C4	1.378 (7)	C22—C23	1.449 (8)
C3—H3	0.9500	C22—H22	0.9500
C4—C5	1.394 (7)	C23—N4	1.356 (6)
C4—H4	0.9500	C23—C24	1.381 (7)
C5—C6	1.379 (7)	C24—C25	1.386 (8)
C5—H5	0.9500	C24—H24	0.9500
C6—C7	1.473 (7)	C25—C26	1.370 (8)
C7—N2	1.269 (6)	C25—H25	0.9500
C7—H7	0.9500	C26—C27	1.419 (7)
N2—C8	1.437 (6)	C26—H26	0.9500
C8—C13	1.393 (8)	C27—N4	1.331 (7)
C8—C9	1.399 (8)	C27—C28	1.509 (8)
C9—C10	1.394 (7)	C28—H28A	0.9800
C9—H9	0.9500	C28—H28B	0.9800
C10—C11	1.380 (8)	C28—H28C	0.9800
C10—H10	0.9500	O1—C29	1.265 (6)
C11—C12	1.397 (8)	O2—C29	1.270 (6)
C11—H11	0.9500	C29—C30	1.490 (8)
C12—C13	1.385 (7)	C30—H30A	0.9800
C12—H12	0.9500	C30—H30B	0.9800
C13—C14	1.517 (7)	C30—H30C	0.9800
C14—C15	1.556 (7)	Cl1—O6	1.422 (4)
C14—H14A	0.9900	Cl1—O5	1.426 (4)
C14—H14B	0.9900	Cl1—O3	1.428 (4)
C15—C16	1.490 (7)	Cl1—O4	1.429 (5)
			
O2—Ni1—O1	63.17 (14)	C16—C15—C14	109.9 (5)
O2—Ni1—N3	96.41 (15)	C16—C15—H15A	109.7
O1—Ni1—N3	158.19 (15)	C14—C15—H15A	109.7
O2—Ni1—N1	164.65 (15)	C16—C15—H15B	109.7
O1—Ni1—N1	103.58 (15)	C14—C15—H15B	109.7
N3—Ni1—N1	97.63 (17)	H15A—C15—H15B	108.2
O2—Ni1—N2	90.38 (15)	C21—C16—C17	116.9 (5)
O1—Ni1—N2	81.85 (16)	C21—C16—C15	123.1 (5)
N3—Ni1—N2	107.24 (18)	C17—C16—C15	120.0 (5)
N1—Ni1—N2	79.57 (16)	C18—C17—C16	121.8 (6)
O2—Ni1—N4	84.69 (15)	C18—C17—H17	119.1
O1—Ni1—N4	90.08 (17)	C16—C17—H17	119.1
N3—Ni1—N4	79.87 (18)	C17—C18—C19	120.6 (6)
N1—Ni1—N4	103.85 (16)	C17—C18—H18	119.7
N2—Ni1—N4	171.81 (19)	C19—C18—H18	119.7
O2—Ni1—C29	31.69 (15)	C20—C19—C18	119.5 (6)
O1—Ni1—C29	31.52 (16)	C20—C19—H19	120.3
N3—Ni1—C29	127.48 (18)	C18—C19—H19	120.3
N1—Ni1—C29	134.88 (19)	C19—C20—C21	120.5 (6)
N2—Ni1—C29	86.64 (18)	C19—C20—H20	119.7
N4—Ni1—C29	85.73 (18)	C21—C20—H20	119.7
C6—N1—C1	116.9 (4)	C20—C21—C16	120.7 (5)
C6—N1—Ni1	111.2 (3)	C20—C21—N3	119.2 (5)
C1—N1—Ni1	131.9 (4)	C16—C21—N3	120.1 (5)
N1—C1—C3	120.6 (5)	C22—N3—C21	115.2 (5)
N1—C1—C2	119.5 (5)	C22—N3—Ni1	110.9 (4)
C3—C1—C2	119.8 (5)	C21—N3—Ni1	133.9 (4)
C1—C2—H2A	109.5	N3—C22—C23	120.6 (5)
C1—C2—H2B	109.5	N3—C22—H22	119.7
H2A—C2—H2B	109.5	C23—C22—H22	119.7
C1—C2—H2C	109.5	N4—C23—C24	123.5 (6)
H2A—C2—H2C	109.5	N4—C23—C22	116.9 (5)
H2B—C2—H2C	109.5	C24—C23—C22	119.6 (5)
C4—C3—C1	121.9 (5)	C23—C24—C25	117.6 (6)
C4—C3—H3	119.1	C23—C24—H24	121.2
C1—C3—H3	119.1	C25—C24—H24	121.2
C3—C4—C5	117.4 (5)	C26—C25—C24	120.0 (6)
C3—C4—H4	121.3	C26—C25—H25	120.0
C5—C4—H4	121.3	C24—C25—H25	120.0
C6—C5—C4	118.6 (5)	C25—C26—C27	119.2 (6)
C6—C5—H5	120.7	C25—C26—H26	120.4
C4—C5—H5	120.7	C27—C26—H26	120.4
N1—C6—C5	124.5 (5)	N4—C27—C26	120.9 (6)
N1—C6—C7	116.0 (5)	N4—C27—C28	120.5 (5)
C5—C6—C7	119.5 (5)	C26—C27—C28	118.6 (6)
N2—C7—C6	120.5 (5)	C27—C28—H28A	109.5
N2—C7—H7	119.8	C27—C28—H28B	109.5
C6—C7—H7	119.8	H28A—C28—H28B	109.5
C7—N2—C8	115.8 (4)	C27—C28—H28C	109.5
C7—N2—Ni1	111.2 (3)	H28A—C28—H28C	109.5
C8—N2—Ni1	129.7 (3)	H28B—C28—H28C	109.5
C13—C8—C9	120.0 (5)	C27—N4—C23	118.6 (5)
C13—C8—N2	120.6 (5)	C27—N4—Ni1	130.4 (4)
C9—C8—N2	119.4 (5)	C23—N4—Ni1	109.1 (4)
C10—C9—C8	120.1 (6)	C29—O1—Ni1	87.8 (4)
C10—C9—H9	119.9	C29—O2—Ni1	88.7 (3)
C8—C9—H9	119.9	O1—C29—O2	120.2 (6)
C11—C10—C9	119.8 (6)	O1—C29—C30	119.3 (6)
C11—C10—H10	120.1	O2—C29—C30	120.5 (5)
C9—C10—H10	120.1	O1—C29—Ni1	60.7 (3)
C10—C11—C12	119.9 (6)	O2—C29—Ni1	59.7 (3)
C10—C11—H11	120.0	C30—C29—Ni1	175.6 (4)
C12—C11—H11	120.0	C29—C30—H30A	109.5
C13—C12—C11	120.8 (6)	C29—C30—H30B	109.5
C13—C12—H12	119.6	H30A—C30—H30B	109.5
C11—C12—H12	119.6	C29—C30—H30C	109.5
C12—C13—C8	119.3 (5)	H30A—C30—H30C	109.5
C12—C13—C14	121.0 (5)	H30B—C30—H30C	109.5
C8—C13—C14	119.7 (5)	O6—Cl1—O5	110.9 (3)
C13—C14—C15	113.1 (5)	O6—Cl1—O3	109.8 (3)
C13—C14—H14A	109.0	O5—Cl1—O3	108.6 (3)
C15—C14—H14A	109.0	O6—Cl1—O4	109.7 (3)
C13—C14—H14B	109.0	O5—Cl1—O4	109.7 (3)
C15—C14—H14B	109.0	O3—Cl1—O4	108.1 (3)
H14A—C14—H14B	107.8		
			
O2—Ni1—N1—C6	43.6 (9)	C15—C16—C21—N3	−3.0 (8)
O1—Ni1—N1—C6	72.7 (4)	C20—C21—N3—C22	56.1 (7)
N3—Ni1—N1—C6	−112.4 (4)	C16—C21—N3—C22	−123.4 (5)
N2—Ni1—N1—C6	−6.2 (4)	C20—C21—N3—Ni1	−122.4 (5)
N4—Ni1—N1—C6	166.2 (4)	C16—C21—N3—Ni1	58.1 (7)
C29—Ni1—N1—C6	68.3 (5)	O2—Ni1—N3—C22	74.9 (4)
O2—Ni1—N1—C1	−136.2 (6)	O1—Ni1—N3—C22	55.2 (6)
O1—Ni1—N1—C1	−107.1 (5)	N1—Ni1—N3—C22	−111.3 (4)
N3—Ni1—N1—C1	67.8 (5)	N2—Ni1—N3—C22	167.3 (3)
N2—Ni1—N1—C1	174.0 (5)	N4—Ni1—N3—C22	−8.5 (4)
N4—Ni1—N1—C1	−13.6 (5)	C29—Ni1—N3—C22	68.1 (4)
C29—Ni1—N1—C1	−111.5 (5)	O2—Ni1—N3—C21	−106.6 (5)
C6—N1—C1—C3	−4.2 (8)	O1—Ni1—N3—C21	−126.3 (5)
Ni1—N1—C1—C3	175.6 (4)	N1—Ni1—N3—C21	67.2 (5)
C6—N1—C1—C2	177.3 (5)	N2—Ni1—N3—C21	−14.2 (5)
Ni1—N1—C1—C2	−2.9 (9)	N4—Ni1—N3—C21	170.0 (5)
N1—C1—C3—C4	3.0 (10)	C29—Ni1—N3—C21	−113.4 (5)
C2—C1—C3—C4	−178.4 (6)	C21—N3—C22—C23	−177.1 (5)
C1—C3—C4—C5	−0.5 (10)	Ni1—N3—C22—C23	1.7 (6)
C3—C4—C5—C6	−0.6 (9)	N3—C22—C23—N4	11.5 (8)
C1—N1—C6—C5	3.2 (9)	N3—C22—C23—C24	−169.8 (5)
Ni1—N1—C6—C5	−176.6 (5)	N4—C23—C24—C25	−2.6 (8)
C1—N1—C6—C7	−178.8 (5)	C22—C23—C24—C25	178.8 (5)
Ni1—N1—C6—C7	1.4 (6)	C23—C24—C25—C26	3.9 (8)
C4—C5—C6—N1	−0.8 (9)	C24—C25—C26—C27	−0.6 (8)
C4—C5—C6—C7	−178.7 (5)	C25—C26—C27—N4	−4.4 (8)
N1—C6—C7—N2	8.9 (8)	C25—C26—C27—C28	173.3 (5)
C5—C6—C7—N2	−173.0 (6)	C26—C27—N4—C23	5.7 (8)
C6—C7—N2—C8	−175.5 (5)	C28—C27—N4—C23	−172.0 (5)
C6—C7—N2—Ni1	−13.9 (7)	C26—C27—N4—Ni1	−156.9 (4)
O2—Ni1—N2—C7	−157.5 (4)	C28—C27—N4—Ni1	25.5 (8)
O1—Ni1—N2—C7	−94.7 (4)	C24—C23—N4—C27	−2.2 (8)
N3—Ni1—N2—C7	105.6 (4)	C22—C23—N4—C27	176.4 (5)
N1—Ni1—N2—C7	10.8 (4)	C24—C23—N4—Ni1	163.8 (4)
C29—Ni1—N2—C7	−126.0 (4)	C22—C23—N4—Ni1	−17.6 (6)
O2—Ni1—N2—C8	0.8 (5)	O2—Ni1—N4—C27	80.4 (5)
O1—Ni1—N2—C8	63.7 (5)	O1—Ni1—N4—C27	17.3 (5)
N3—Ni1—N2—C8	−96.0 (5)	N3—Ni1—N4—C27	177.9 (5)
N1—Ni1—N2—C8	169.2 (5)	N1—Ni1—N4—C27	−86.7 (5)
C29—Ni1—N2—C8	32.3 (5)	C29—Ni1—N4—C27	48.6 (5)
C7—N2—C8—C13	−105.4 (6)	O2—Ni1—N4—C23	−83.4 (3)
Ni1—N2—C8—C13	97.1 (6)	O1—Ni1—N4—C23	−146.5 (3)
C7—N2—C8—C9	73.2 (7)	N3—Ni1—N4—C23	14.1 (3)
Ni1—N2—C8—C9	−84.3 (6)	N1—Ni1—N4—C23	109.5 (3)
C13—C8—C9—C10	1.3 (8)	C29—Ni1—N4—C23	−115.2 (4)
N2—C8—C9—C10	−177.3 (5)	O2—Ni1—O1—C29	−2.3 (3)
C8—C9—C10—C11	0.4 (8)	N3—Ni1—O1—C29	19.7 (6)
C9—C10—C11—C12	−0.7 (9)	N1—Ni1—O1—C29	−174.0 (3)
C10—C11—C12—C13	−0.6 (9)	N2—Ni1—O1—C29	−96.9 (3)
C11—C12—C13—C8	2.3 (8)	N4—Ni1—O1—C29	81.7 (3)
C11—C12—C13—C14	−178.3 (5)	O1—Ni1—O2—C29	2.3 (3)
C9—C8—C13—C12	−2.6 (8)	N3—Ni1—O2—C29	−169.6 (3)
N2—C8—C13—C12	176.0 (5)	N1—Ni1—O2—C29	34.2 (8)
C9—C8—C13—C14	177.9 (5)	N2—Ni1—O2—C29	83.0 (3)
N2—C8—C13—C14	−3.5 (8)	N4—Ni1—O2—C29	−90.5 (3)
C12—C13—C14—C15	111.2 (6)	Ni1—O1—C29—O2	3.9 (5)
C8—C13—C14—C15	−69.3 (7)	Ni1—O1—C29—C30	−175.0 (5)
C13—C14—C15—C16	148.2 (5)	Ni1—O2—C29—O1	−4.0 (5)
C14—C15—C16—C21	−117.4 (6)	Ni1—O2—C29—C30	174.9 (5)
C14—C15—C16—C17	61.1 (6)	O2—Ni1—C29—O1	176.1 (5)
C21—C16—C17—C18	0.9 (8)	N3—Ni1—C29—O1	−170.9 (3)
C15—C16—C17—C18	−177.7 (5)	N1—Ni1—C29—O1	8.2 (4)
C16—C17—C18—C19	−0.2 (9)	N2—Ni1—C29—O1	79.9 (3)
C17—C18—C19—C20	−0.4 (9)	N4—Ni1—C29—O1	−97.1 (3)
C18—C19—C20—C21	0.2 (9)	O1—Ni1—C29—O2	−176.1 (5)
C19—C20—C21—C16	0.5 (9)	N3—Ni1—C29—O2	13.0 (4)
C19—C20—C21—N3	−179.0 (5)	N1—Ni1—C29—O2	−167.9 (3)
C17—C16—C21—C20	−1.0 (8)	N2—Ni1—C29—O2	−96.2 (3)
C15—C16—C21—C20	177.5 (5)	N4—Ni1—C29—O2	86.8 (3)
C17—C16—C21—N3	178.4 (5)		

### Hydrogen-bond geometry (Å, °)

**Table:**

*D*—H···*A*	*D*—H	H···*A*	*D*···*A*	*D*—H···*A*
C4—H4···O1^i^	0.95	2.45	3.274 (6)	144
C7—H7···O4^ii^	0.95	2.41	3.290 (7)	155
C20—H20···O3^iii^	0.95	2.43	3.366 (8)	168
C15—H15B···O2	0.99	2.54	3.523 (7)	174
C15—H15B···N3	0.99	2.47	2.935 (7)	108

Symmetry codes: (i) −*x*+2, −*y*+2, −*z*+1; (ii) *x*+1, *y*+1, *z*; (iii) *x*+1, *y*, *z*.
